# Overview of the Potential Role of *Malassezia* in Gut Health and Disease

**DOI:** 10.3389/fcimb.2020.00201

**Published:** 2020-05-26

**Authors:** Madeleine Spatz, Mathias L. Richard

**Affiliations:** Université Paris-Saclay, INRAE, AgroParisTech, Micalis Institute, Jouy-en-Josas, France

**Keywords:** mycobiota, inflammatory bowel diseases, gut, cancer, *Malassezia*

## Abstract

*Malassezia* is the most prevalent fungus identified in the human skin microbiota; originally described at the end of the nineteenth century, this genus is composed of at least 14 species. The role of *Malassezia* on the skin remains controversial because this genus has been associated with both healthy skin and pathologies (dermatitis, eczema, etc.). However, with the recent development of next-generation sequencing methods, allowing the description of the fungal diversity of various microbiota, *Malassezia* has also been identified as a resident fungus of diverse niches such as the gut or breast milk. A potential role for *Malassezia* in gut inflammation and cancer has also been suggested by recent studies. The aim of this review is to describe the findings on *Malassezia* in these unusual niches, to investigate what is known of the adaptation of *Malassezia* to the gut environment and to speculate on the role of this yeast in the host physiology specifically related to the gastrointestinal tract.

## Introduction

*Malassezia* is the major component of the fungal skin microbiota of various mammals, representing more than 90% of the fungal population in most skin niches (Dawson, 2019). As such, *Malassezia* yeasts have been associated with human skin disorders (Gupta et al., 2004) such as dermatitis (Gaitanis et al., 2008; Darabi et al., 2009; Barac et al., 2015), pityriasis versicolor (Magiatis et al., 2013), or dandruff (Gemmer et al., 2002). The mechanisms by which *Malassezia* cells trigger such diseases are not yet clearly identified, but the current hypothesis is that the diseases can be induced either by direct invasion of the tissue by fungal filaments or indirectly through immunological and metabolic mechanisms induced by the yeast. While one can consider that *Malassezia* is perfectly adapted to the skin environment, increasing data suggest that this genus can also be found in other body compartments (Theelen et al., 2018). The development of next-generation sequencing (NGS) methods have overcome the problems associated with culture-based methods, allowing the identification of *Malassezia* in surprisingly diverse localizations, such as the digestive tract, or in very unexpected locations with the identification of *Malassezia*-like organisms in deep-sea vents, for instance (Amend, 2014). The gut microbiota is not only composed of microorganisms such as bacteria, archaea, viruses, and protozoans but also colonized by numerous fungal cells (Sekirov et al., 2010). The role of fungal gut microbiota in host homeostasis, as well as in several physio-pathologic settings, is now clearly identified (Sekirov et al., 2010; Limon et al., 2017; Richard and Sokol, 2019). However, many questions remain regarding the specific fungal species involved in this role. In this review, we will focus on recent studies that described the identification of *Malassezia* in various non-skin environments, and we will particularly concentrate on the potential role of *Malassezia* in gut health and diseases (Chen et al., 2011; Hamad et al., 2012; Gouba et al., 2013; Suhr et al., 2016; Hallen-Adams and Suhr, 2017).

## Identification of *Malassezia* in Niches Unrelated to the Skin

Although it has been long regarded as a strictly skin-located fungus, *Malassezia* is now regularly identified in various samples using NGS methods. The culture of *Malassezia* is known to be difficult, and this is probably a reason for the very low level of *Malassezia* identified using culture-dependent methods under conditions where this genus is not highly abundant. With culture-independent methods, the unexpected diversity of fungi has been unraveled, and *Malassezia* strains have been identified in unforeseen environments. Thus, *Malassezia* strains have been found in diverse localizations such as murine (Limon et al., 2019) and human (Suhr et al., 2016; Hallen-Adams and Suhr, 2017) gut, human breast milk (Boix-Amorós et al., 2017), and internal organs, including the central nervous system (Alonso et al., 2017, 2018a,b). Moreover, *Malassezia* DNA has also been found in more diverse ecosystems, such as marine environments (Amend, 2014), but this field is beyond the scope of this review, although it opens a very interesting research axis. Indeed, culture-independent studies highlighted that *Malassezia* DNA nearly identical to human-associated *Malassezia* DNA can be found in various habitats (Amend, 2014), such as in deep-sea sediments, in corals, in lobster guts or on the exoskeleton of nematodes, but more sequences and taxonomic studies are needed to allow a complete understanding of the *Malassezia* genus and its distribution on earth.

Before describing the data gathered so far on *Malassezia* identification in niches unrelated to the skin it is important to raise the question of the samples contamination. Fungi represent 30% of the skin microbiota and *Malassezia* strains are by far the majority on the skin, consequently representing a probable cause of samples contamination either during the sampling or of the samples afterward in the lab. These questions have been raised by some authors in their study and they provided several solutions:

- Contamination after sampling: This is the easiest problem to solve and a problem common to all NGS experiments. Mostly the experimentators will use internal controls, and sequenced them, like the “reagent-only” controls, but one can also see particular attention directed to the tip used, the room for extraction, the disposable supplies, etc. (Alonso et al., 2018a). Thus, the global microbial contamination would be fairly reduced and contaminating sequences can be removed from the sequences upstream the analyze pipeline.
- contamination during sampling: That matter on the other hand is much more difficult to work around. The main solutions that have been used in the experiments are: (i) the way of preparing the area before sampling like cleaning thoroughly the area (breast, rectum, etc.) prior to sample collection (Boix-Amorós et al., 2019), or (ii) getting samples directly from explant in an areas that were never in close contact with the skin like getting the mucosa-associated microbiota or from the organ itself (Liguori et al., 2016; Aykut et al., 2019). This matter, however, has not been strictly tackled especially regarding *Malassezia* in non-skin niches which is not totally surprising since this is a young area of research, but this is certainly an area of improvement.

Thus, we need to keep in mind that, a part of *Malassezia* identified might comes from the skin during samples collection however, we can also consider the fact that from the numerous reports, from very different sampling method, the identification of *Malassezia* by numerous laboratories is very unlikely to be only sample contamination. However, we will see further in this review that colonization via the skin is a very likely explanation of how *Malassezia* first enter the different niches.

### Malassezia in Human Breastmilk

An analysis of the composition of the fungal microbiota present in the human breastmilk revealed two main pieces of information: (i) fungi are much more abundant in milk than bacteria compared to what is observed in many other niches. Indeed, fungi represent 30% of the total skin microbial population, while fungi represent ~0.1% in the gut for instance. (ii) Among the fungal strains, more than 40% of the total genera identified by pyrosequencing are represented by *Malassezia* strains (Boix-Amorós et al., 2017, 2019). Interestingly, this colonization was identified worldwide with samples from different continents (Spain, China, South Africa, and Finland). *Malassezia* strains, particularly *M. globosa* and *M. restricta*, were detected in all the samples analyzed (Boix-Amorós et al., 2017). The authors ruled out the possibility that their samples were contaminated by the skin microbiota but speculated that the primary source of *Malassezia* in breast milk might be the possible transfer of fungi from the skin surrounding the breast or from the baby's mouth or skin to the breast milk. To date, no studies have followed the milk microbiota from the early production of the milk to later on in order to elaborate a possible route of colonization.

### Malassezia in the Central Nervous System

In the last 3 years, a single team has raised the possibility of the involvement of fungi in different neurologic diseases, such as Alzheimer's disease, multiple sclerosis, or amyotrophic lateral sclerosis (Alonso et al., 2017, 2018a,b). Very surprisingly, using NGS methods, Alonso and coworkers identified fungi and bacteria in the brain tissue of healthy subjects and of patients and showed that in some cases, specific fungal strains seemed more abundant in the affected brains than in the brains from healthy donors. No data in these publications are provided on the actual quantities of these microorganisms in the brain, and this information would be important to have in the future. Interestingly, among the fungal strains identified, a large percentage of samples showed the presence of DNA from *Malassezia* strains, suggesting possible colonization by *Malassezia* in the central nervous system of patients (Alonso et al., 2018b). Very importantly these data have to be confirmed by independent teams, along with the addition of quantitative data in order to evaluate a potential causal link between this *Malassezia* colonization and the diseases that has not yet been proven.

### Malassezia in Other Human Body Sites

The presence of *Malassezia* in different human niches in healthy patients is controversial, particularly its colonization of the mouth (Ghannoum et al., 2010; Dupuy et al., 2014). On the other hand, in infection contexts, *Malassezia* strains have been more regularly identified in blood, urine, vagina, and lung (Theelen et al., 2018). For instance, *M. furfur* (Kaneko et al., 2012; Iatta et al., 2018) was identified in blood and in central venous catheters in 4% of neonate patients.

### Malassezia as a Member of the Gut Microbiota From Healthy Subjects

In 2017, Dawson, Boekhout and coworkers asked in their review whether *Malassezia* strains were indeed part of the gut microbiota (Theelen et al., 2018). Three years later, an increasing number of publications reported the identification of *Malassezia* in healthy fecal samples (Chen et al., 2011; Hamad et al., 2012; Gouba et al., 2013; Suhr et al., 2016; Hallen-Adams and Suhr, 2017). At steady state, the *Malassezia* genus has been reported to be the second most abundant genus among all human stools analyzed by internal transcribed spacer (ITS)-gene sequencing in proportions from 2 to 4% (Gouba et al., 2013; Suhr et al., 2016; Raimondi et al., 2019), while *M. restricta* (Suhr et al., 2016; Nash et al., 2017; Auchtung et al., 2018) (more than 80% and can reach 3.8% of the total abundance Raimondi et al., 2019) and *M. globosa* (Nash et al., 2017) (36%) represent the most abundant species. Moreover, *M. pachydermatis* (Chen et al., 2011; Hamad et al., 2012; Gouba et al., 2013), *M. restricta* (Hamad et al., 2012; Gouba et al., 2013), and *M. globosa* (Hamad et al., 2012; Gouba et al., 2013) were identified by molecular detection (specific primers 18S Chen et al., 2011; Hamad et al., 2012 or JPD1/JDP2 Gouba et al., 2013) and by culture media isolation (Dixon agar medium Gouba et al., 2013). The presence of *Malassezia* living cells and a large amount of *Malassezia* DNA found in the gut content have also been associated with the intestinal mucosa (Liguori et al., 2016), which strongly suggested that this genus has the capacity to at least survive in the intestinal environment. The growth or at least survival of *Malassezia* suggested that some *Malassezia* strains found favored culture conditions within the intestine.

## Diversity and Growth Conditions of *Malassezia*

As mentioned above, *Malassezia* strains are mostly found on the skin of humans (Findley et al., 2013) and mammals (Cabañes et al., 2007; Velegraki et al., 2015) suggesting specific growth conditions *in vivo*: temperature of ~33°C, aerobic conditions, and low nutrient availability. However, with the identification of *Malassezia* strains in niches very different from the skin (see Table 1), such as the gut, questions have arisen regarding how this fungus can adapt to such various environmental conditions of growth.

**Table 1:** Compartments where *Malassezia* were identified from and the percentage of the fungal microbiota.

**Compartments**	***Malassezia* (% of fungal microbiota)**
Brain	0.5 (Alonso et al., 2018b)
Gut	2–4 (Gouba et al., 2013; Suhr et al., 2016; Raimondi et al., 2019)
Lung	No data available in healthy subject
Milk	20–50 (Boix-Amorós et al., 2019)
Mouth	13–96 (Dupuy et al., 2014)
Skin	50–80 (Dawson, 2019)
Urine	No data available
Vagina	No data available

### Malassezia Growth on the Skin

The *Malassezia* genus contains at least 14 species (Gaitanis et al., 2012; Dawson, 2019), among which *M. globosa, M. restricta, M. sympodialis*, and *M. furfur* are the most commonly identified species (Ashbee, 2007; Tajima et al., 2008) and *M. pachydermatis* is the only lipid-independent fungus (Ashbee, 2007). Indeed, one of the main physiological traits of this genus is the inability of almost all *Malassezia* strains to synthesize fatty acids (Shifrine and Marr, 1963) *de novo*. Consequently, *in vivo, Malassezia* strains require an external source of lipids for growth. This dependency is not a problem on the skin; indeed, skin epithelium layers can produce lipid-rich sebum mostly from sebaceous glands, which will provide a suitable source of long-chain fatty acids for optimal *Malassezia* growth (Gaitanis et al., 2012). Consequently, *Malassezia* has developed a large set of enzymes that digest these compounds with lipases and phospholipases (Velegraki et al., 2015).

This physiological constraint can explain the distribution of *Malassezia* on the body surface: sebum-rich areas such as the head, arms, legs, and torso are rich in *Malassezia* strains, while the other areas of the body show lower abundance. More specifically, *M. restricta* is predominant on the forehead and inside and outside of the ears, and *M. globosa* is predominant on the back, occiput, and groin (Theelen et al., 2018). Accordingly, on the foot, which is a very different environment by many parameters (low level of sebum, humidity, skin type, etc.), the colonization of fungi is much more diverse. As a consequence, *Malassezia* is not the predominant genus in the foot (Byrd et al., 2018), and the foot fungal community seems much more variable over time; this result possibly explains the higher frequency of fungal infections observed in environments with no true homeostasis, leaving colonization chances for opportunistic microorganisms (Findley et al., 2013).

### From the Human Skin to the Gut

These observations do not explain the possible colonization of the intestinal tract by *Malassezia* strains. The first question is the source of colonization. As observed above, *Malassezia* represents a large proportion of the breastmilk mycobiota. Indeed, human milk is composed of 39 g/L fat (Jensen, 1999), and the majority of lipids are triglycerides and fatty acids. We can assume that *Malassezia* strains can use these lipid sources for their own growth, even if no clear correlation was found between the *Malassezia* genus and fat (Boix-Amorós et al., 2017). We can consider that the breast milk can be one of the entry points during primo-colonization, but breastfeeding in the world is far below 50%, so this cannot be the only explanation (Victora et al., 2016). Logically since *Malassezia* is in high concentration on the skin, we can hypothesize that simple transfer from our own skin microbiota can be part of the colonization process.

Thus, the actual survival or even development of *Malassezia* cells within the gut remains a source of interrogations. Within the gut, three main parameters differ from the skin: (i) the absence of sebum, the source of lipids, (ii) the higher temperature, and (iii) the low level of oxygen.

#### Lipids

One can consider that the absence of sebum should not be a major problem since many other sources of lipids are available along the gut. For instance, bile acids synthesized by hepatocytes and stored in the gallbladder as bile salts are regularly poured into the intestine to allow the emulsion of fat, making lipids available for host cells and microorganisms. *Malassezia* fungi can thus use this source of lipids. Thus, *Malassezia* strains should be able to obtain enough sources of lipids within the gut for their growth using molecules coming from the diet or from other microorganisms in the intestinal microbiota.

#### Temperature

While the temperature for *Malassezia* growth is often considered to be restricted to the skin temperature (33°C), *Malassezia* isolated from hair follicles and sebaceous glands can grow in an environment slightly different from the surface of the cutaneous layer, with a slightly higher temperature and lower oxygen concentration. In accordance with this observation, numerous data have shown that most *Malassezia* strains can grow at 37°C or above, with a maximum temperature identified at ~40–41°C (Gaitanis et al., 2012).

#### Oxygen

For microorganisms, a low oxygen concentration can be a potent inhibitor of growth, but again, this might not represent a strong challenge for *Malassezia* strains. The level of oxygen is not constant along the intestine but follows a decreasing gradient from the mouth to the rectum (Espey, 2013); thus, *Malassezia* might find parts of the gut with suitable conditions for development. As stated above, the observation of growth in follicles and sebaceous glands supports a certain flexibility in terms of the levels of oxygen needed for *Malassezia*.

Reports on the growth capacity of *Malassezia* strains in anaerobic conditions are sparse and do not give a general or specific overview for each species. The most often cited work is a publication in 1981 in *Sabouraudia* from two Sweden researchers, Faergemann and Bernander, describing the microaerophilic and anaerobic growth of *Pityrosporum* species (Faergemann and Bernander, 1981). *Pityrosporum* and *Malassezia* were two names given to the same fungi before *Malassezia* was chosen in 1996 (Dolenc-Voljč, 2017). In this study (Faergemann and Bernander, 1981), the authors tested only *M. furfur, M. sympodialis*, and *M. pachydermatis* in different media and with different levels of oxygen. They concluded that the strains were able to grow in microaerophilic and anaerobic conditions but that anaerobic growth was much weaker with slow growth and very small colonies. However, a recent study reported that *M. pachydermatis* is unable to grow under anaerobic conditions, which contradicted the data from the Swedish team suggesting that *M. pachydermatis* might have the capacity to grow under these conditions but that the growth is clearly dependent on many environmental factors that we do not master and understand yet (Tylicki et al., 2008) and can also simply depends on strains variability. It is well-known that it is difficult to grow *Malassezia* strains *in vitro* under laboratory conditions. In addition, as *Malassezia* form aggregates and grow better when they are not isolated on a plate, the evaluation of the CFU as well as using counting cells under the microscope is almost impossible. Altogether, this probably explains the lack of specific and quantitative information about growth under anaerobic conditions.

Youngchim et al., while studying the hyphae formation of *M. furfur*, used microaerophilic conditions (anaerobic jar) for the induction of morphogenesis, but no data are available on the growth rate under these conditions (Youngchim et al., 2013). However, it seems that low levels of oxygen induce *Malassezia* morphogenesis, which is an interesting feature that can participate in the colonization of this strain in the human epithelium. Indeed, germ tubes and hyphae are known to be penetrating structures that can help *Malassezia* cross epithelial barriers (Tati et al., 2016). Consequently, *Malassezia* hyphae might help fungal cells reach areas rich in nutrients over the highly keratinized top layers of the skin (Brand, 2012).

Altogether, these data suggest that although *Malassezia* mainly localizes with the skin microbiota, this fungal microorganism has the capacity to at least survive within the gut. However, the resistance to acidic pH, for example, has not been clearly documented, so thorough characterization of each *Malassezia* species is still needed.

## Fungal Gut Microbiota

In the past few years, the gut microbiota has become a key player in human health studies. When the gut microbiota is described, it tends to be reduced to its bacterial population only. Indeed, bacteria represent ~99.1% of the population established in the colon (Qin et al., 2010). However, technological advances, such as NGS, have allowed deeper investigation of the microbial population and have revealed that the microbiota is also populated by archaea, viruses, protozoans and 0.01–0.1% fungi (Qin et al., 2010; Huffnagle and Noverr, 2013; Nash et al., 2017). In addition, changes in the composition of fungal gut microbiota, the mycobiota, have been associated with several gut-related diseases, such as inflammatory bowel disease (IBD) (Chehoud et al., 2015; Liguori et al., 2016; Sokol et al., 2017), irritable bowel syndrome (Botschuijver et al., 2017), colorectal cancer (Gao et al., 2017), and alcoholic liver disease (Yang et al., 2017).

### Fungal Gut Colonization

The current theory is that most fungi transit through the digestive tract without being able to implant. As with bacteria, fungi can primo-colonize the intestine at birth (Bliss et al., 2008; Nagata et al., 2012) and during breastfeeding (Nagata et al., 2012; Boix-Amorós et al., 2017); then, they can simply be brought by food, the respiratory tracts or the contacts between the mouth and the skin (Schulze and Sonnenborn, 2009; Koh, 2013). Indeed, on the skin, fungi represent 5–10% (Byrd et al., 2018) of the microbial population, consequently representing a large reservoir for colonization. Additionally, food is an important source of fungi, which play an important role in the transformation processes during food preparation and can be ingested in large quantities. There is no doubt, however, that there are huge differences between continents or even countries depending on their food cultural habits. Nevertheless, it has been shown that some species can survive in this specific environment and manage to adhere to human epithelial cells more effectively and thus persist in the intestine, which is a characteristic of the *Candida* genus, such as *C. albicans* (Raimondi et al., 2019). The fact that a strain can persist or only transit in the body does not account for its capacity to influence host health. A good example is the positive effect of *Saccharomyces boulardii* CNCM I-745 on antibiotic associated diarrhea and acute gastroenteritis, since *S. boulardii* is known to be unable to settle in the human gut but is cleared in 2 to 5 days (Buts and De Keyser, 2006).

The mycobiota appears to be relatively stable along the digestive tract: from 10^3^ (Darabi et al., 2009) in the stomach to up to 10^5–6^ (Gaitanis et al., 2008; Magiatis et al., 2013) microorganisms per gram of content in the colon (Slmon and Gorbach, 1984). The bacterial microbiota abundance increased dramatically from the stomach to the colon, from 10^2^ (Gupta et al., 2004) in the stomach to up to 10^12^ (Richard and Sokol, 2019) microorganisms per gram of content in the colon (Slmon and Gorbach, 1984). As such, the fungal to bacterial cell abundance ratio is variable from the stomach to the colon and can be much more favorable to fungi in the upper digestive tract. Further investigations are thus needed in these compartments where fungi may have a stronger influence on host health.

### Composition and Diversity of the Mycobiota

The composition of the mycobiota, as the rest of the gut microorganisms, varies according to the environment (Suhr et al., 2016), diet (Hoffmann et al., 2013; David et al., 2014; Hallen-Adams and Suhr, 2017; Heisel et al., 2017; Yang et al., 2017), sex (Markle et al., 2013; Strati et al., 2016; Borges et al., 2018), and health of the host (Richard et al., 2015; Nash et al., 2017; Sokol et al., 2017). There are two major approaches to investigate the gut mycobiota: culture-dependent (Gouba et al., 2013; Becker et al., 2014; Borges et al., 2018) or culture-independent methods (Qin et al., 2010; Donovan et al., 2018). Culture-dependent methods have the strong advantage of resulting in a microorganism that can be used directly either in interactions with cells or for metabolite production, for instance. However, this method allows only the isolation of a very low percentage of living organisms in samples for simple technical reasons: the media, pH, oxygenation, or temperature may not be optimized. On the other hand, culture-independent methods are much more efficient for the identification of a large percentage of the microorganisms present with fewer technical constraints. To analyze the fungal population with a culture-independent method, the major targets are the internal transcribed sequences (ITSs) ITS1 and ITS2 (Huffnagle and Noverr, 2013; Tang J. et al., 2015; Wang et al., 2015; Donovan et al., 2018; Yang et al., 2018). These regions are highly divergent between fungi and can even allow identification to the fungal species level; for further information, refer to Richard and Sokol (2019). However, some strains have been identified by culture-dependent methods and not by culture-independent methods (Hamad et al., 2017; Richard and Sokol, 2019), revealing that both methods show advantages and limitations (Richard and Sokol, 2019) and that they remain complementary for the exhaustive identification of the intestinal mycobiota.

From these diverse techniques, we have concluded so far that the major phyla of the intestinal mycobiota are Ascomycota and Basidiomycota (Suhr et al., 2016; Nash et al., 2017; Borges et al., 2018; Raimondi et al., 2019), with a much lower abundance of Zygomycetes (Borges et al., 2018). The diversity of the mycobiota is relatively low, since 10 genera and 20 different species are usually identified within a healthy individual: *Candida, Saccharomyces, Malassezia, Penicillium, Aspergillus, Debaryomyces, Trichosporon, Galactomyces, Cryptococcus*, and *Cladosporium* (Strati et al., 2016; Suhr et al., 2016; Nash et al., 2017; Auchtung et al., 2018; Raimondi et al., 2019; Richard and Sokol, 2019). With culture media methods from feces, mostly *Candida* (Strati et al., 2016; Borges et al., 2018; Raimondi et al., 2019) and *Saccharomyces* (Strati et al., 2016; Borges et al., 2018; Raimondi et al., 2019) as well as *Debaryomyces* (Raimondi et al., 2019)*, Penicillium* (Strati et al., 2016; Borges et al., 2018)*, Malassezia*, and *Aspergillus* (Strati et al., 2016; Borges et al., 2018; Raimondi et al., 2019) have been identified.

### Role of Fungal Gut Microbiota in Diseases

As previously stated, the increase in the number of studies on the mycobiota revealed changes in the mycobiota composition associated with intestinal diseases. The causality is still to be proven in most of the studies, but it is difficult to consider that this association is simple coincidence. It is probably a sum of different modifications that trigger and enhance the disease with a vicious circle effect.

In the IBD context, mycobiota in the feces and associated with the mucosa have been studied in various cohort patients, showing a clear modification of the fungal community during intestinal inflammation. *In vivo* studies in mouse models have also reinforced the hypothesis that fungi are directly or indirectly involved in IBD symptoms (Tang C. et al., 2015; Wang et al., 2016). From the various studies published to date, in IBD patients, fungal load was increased during flare (Liguori et al., 2016), showing a modification of the equilibrium between Ascomycota and Basidiomycota with a decrease in Ascomycota and an increase in Basidiomycota (Sokol et al., 2017). At the genus and species levels, *Candida* (Chehoud et al., 2015; Liguori et al., 2016) (particularly *C. glabrata* Liguori et al., 2016, *C. tropicalis* (Hoarau et al., 2016), *C. albicans* Standaert-Vitse et al., 2009, *C. utilis* Chehoud et al., 2015, and *C. parapsilosis* Chehoud et al., 2015) was the genus showing an increase in the vast majority of the studies, and *Saccharomyces* was decreased (Hoarau et al., 2016; Liguori et al., 2016; Sokol et al., 2017). Some studies also identified variations in the relative abundance of *Malassezia* with an increase in *M. globosa* (Liguori et al., 2016) and a decrease in *M. sympodialis* (Sokol et al., 2017) (see next section).

In irritable bowel syndrome (IBS), to date, only one team has specifically made a link between the fungal gut community and IBS symptoms. Botschuijver et al. compared 3 groups of 19+/−1 subjects of healthy controls, hypersensitive IBS and normosensitive IBS patients (Botschuijver et al., 2017) and showed a decrease in diversity in both IBS groups; the *Saccharomyces* and *Candida* genera represented two-thirds or more of the mycobiota in these patients, and they represented ~57% in the healthy controls. Additionally, an increase in *Kazachstania turicensis* was one marker of IBS in these specific cohorts. Further analyses and clinical trials are needed to confirm this hypothesis.

In alcoholic liver diseases, the mycobiota is very strongly modified with a drop in diversity in patients, in which *Candida* seems to replace all other fungal genera within the intestinal content. Interestingly, a previous study demonstrated *in vivo* in mice that the use of antifungal drugs can improve alcohol-induced liver injury (Yang et al., 2017). Again, even if this does not prove causality, the authors made an interesting link between the increase in alcohol in the gut, triggering at the same time an increase in gut permeability and fungal burden: the direct consequence was an increase in circulating **ß**-glucans in the blood reaching the liver and triggering liver injuries via IL-1**ß** and Kupffer cells.

Finally, recently, there has been increasingly more data indicating a potential role of the fungal microbiota during the course of colorectal cancer (CRC) or colitis-associated cancer (CAC). Two reports were published in 2019 on the role of Card9 in the regulation of the fungal burden, myeloid-derived suppressor cell expansion and inflammasome activation, allowing the restriction of CRC or CAC development (Malik et al., 2018; Wang et al., 2018). CARD9 indeed participates in the recognition of microorganisms, especially fungi, through several receptors, such as Mincle, NOD2, and Dectin, and thus orchestrates an important part of the host response against fungi from simple overgrowth to deep infection (Richard et al., 2015).

Additionally, in 2019, chitooligosaccharides were shown to prevent the development of CAC through their effect on the balance between bacterial and fungal microbiota (Wu et al., 2019). In addition to these results, several other publications, which are presented in the next chapter, described the potential role of *Malassezia* strains in cancer development.

## *Malassezia* Influences Gut Health

**Figure 1:**
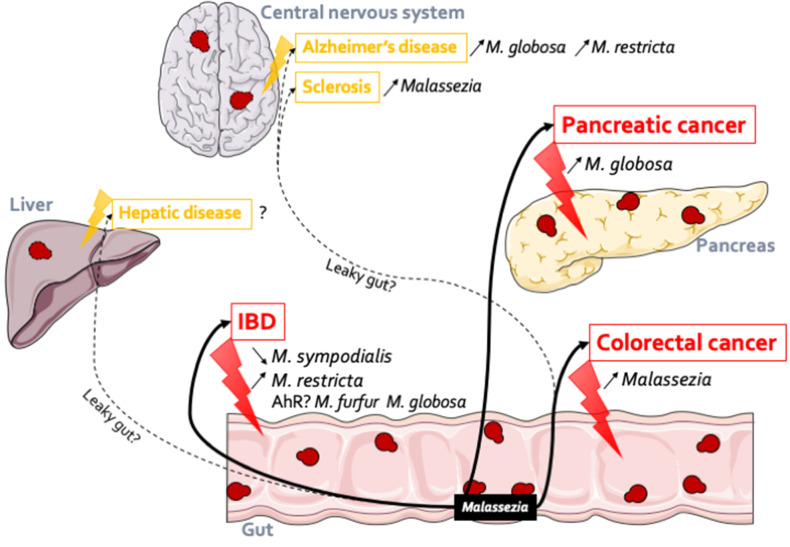
*Malassezia* in the gut: association with several diseases. *Malassezia* strains have an action within the gut. In IBD, *M. sympodialis* has been identified both in flare (Sokol et al., 2017) (decreased) and in remission (Liguori et al., 2016) (increased) of Crohn's disease patients; *M. restricta* (Limon et al., 2019) participates to the production of inflammatory factors and can so exacerbates severe colitis. These observations might be linked to the gene expression regulations through AhR. *Malassezia* genus is more abundant in patients with polyp and colorectal cancer (Gao et al., 2017; Coker et al., 2019). In pancreatic cancer (Aykut et al., 2019), in both patients and mice model, *Malassezia* was increased. *Malassezia* fungi can be implicated in other diseases related or not to the gut. Indeed, their DNA were identified in the central nervous system of Alzheimer's patients (Alonso et al., 2018b) (*M. globosa* and *M. restricta*) and in both multiple sclerosis (Alonso et al., 2018a) and amyotrophic lateral sclerosis patients (Alonso et al., 2017). In the same line we can hypothesis that *Malassezia* could be implicated in hepatic disease due to the liver-gut axis but to date no data confirm this hypothesis.

As highlighted earlier in this review, although a well-described resident of the skin, *Malassezia*, appears to be a prominent component of the gut mycobiota, numerous studies have identified *Malassezia* in fecal samples through culture-dependent and culture-independent methods (Chen et al., 2011; Hamad et al., 2012; Gouba et al., 2013; Suhr et al., 2016; Hallen-Adams and Suhr, 2017). *Malassezia* has been reported in healthy volunteers as a major genus, reaching up to 4% of the total abundance (Gouba et al., 2013; Suhr et al., 2016; Raimondi et al., 2019). *M. restricta* (Hamad et al., 2012; Gouba et al., 2013; Suhr et al., 2016; Nash et al., 2017; Auchtung et al., 2018), *M. globosa* (Hamad et al., 2012; Gouba et al., 2013; Nash et al., 2017), *M. pachydermatis* (Chen et al., 2011; Hamad et al., 2012; Gouba et al., 2013), and *M. sympodialis* (Nash et al., 2017) are the main species that can be found in the gastrointestinal tract. However, these previous studies made very few cases of this presence, mostly considering it transient and with no effect on the host. It is only very recently that *Malassezia* strains have been specifically identified in association with gut diseases and possibly other types of diseases (Figure 1).

### The Impact of Malassezia on IBD

The *Malassezia* genus has only very recently been associated with IBD, both in patients and in mouse models. IBD is composed of two types of disease: Crohn's disease (CD) and ulcerative colitis (UC). Both are characterized by inflammation of the wall of the digestive tract, from the mouth to the rectum for CD and only for the colon for UC (Seyedian et al., 2019).

Two studies highlighted the potential role of *Malassezia* in the development of IBD in the last 3 years: (i) our study while characterizing the global modifications of the bacterial and fungal microbiota for UC and CD patients (Sokol et al., 2017); (ii) and Limon and coworkers in a specific study focused on CD patients (Limon et al., 2019).

In a work where we compared a cohort of 235 IBD patients (UC, CD, flare, or remission) to 38 healthy subjects, our team found that the relative abundance (percentage of reads) of *Malassezia* increased globally during IBD flare. Interestingly, we also showed that *Malassezia* had a negative correlation with many bacteria, especially in UC patients, something that we did not see in other types of disease or during CD. Finally, *M. sympodialis* was negatively correlated with the Dectin1 SNP associated with medically refractory UC (rs2078178, “T” allele12). Altogether, these data from human samples suggest a potential role of *Malassezia* in IBD with possible opposite effects between species.

Using human samples from CD patients (no data on UC) and *in vivo* experiments with a mouse model of colitis, Underhill's team was able to highlight a possible relationship between gut inflammation and the presence of *M. restricta*. Through amplicon-based analysis of the mycobiota of CD patients compared to healthy subjects, *M. restricta* was found to be enriched in the mycobiota-associated mucosa of CD patients (Limon et al., 2019). In addition, in a model of dextran sodium sulfate (DSS)-induced colitis, germ-free or wild-type mice colonized by *M. restricta* showed a worse disease activity index with shorter colons, suggesting that the addition of *M. restricta* alone exacerbated the severity of colitis. However, in their model, *C. albicans*, a well-known pro-inflammatory fungus did not trigger stronger inflammation than the non-treated mice, underlying the complexity of these effects, probably due to the composition of the basal mouse microbiota. The authors also showed a link between *M. restricta* and Card9 signaling: the Card9-S12N polymorphism in CD patients which was strongly linked to the presence of *Malassezia* spp. Using CARD9KO mice and colonization with *M. restricta*, the authors suggested that the pro-inflammatory effects (cytokine production, colitis symptoms) due to *M. restricta* were dependent on Card9.

A possible mechanism explaining the effect of *Malassezia* strains on IBD development can be the link between these fungi and the aryl hydrocarbon receptor (AhR). Indeed, the majority of *Malassezia* strains, especially *M. furfur* (Gaitanis et al., 2008) and *M. globosa* (Magiatis et al., 2013), are capable of synthesizing indole ligands that can act on AhR (Gaitanis et al., 2008; Magiatis et al., 2013; Furue et al., 2014; Wheeler et al., 2017). AhR is a cytoplasmic transcriptional regulator found not only in epithelial cells such as skin cells but also in many other cell types throughout the body (Lamas et al., 2018), and AhR has numerous endogenous ligands with opposite effects on cell functions, generating a very complex network of regulation that has not been completely elucidated. AhR is involved in many functions, including the regulation of the expression of enzymes involved in xenobiotic metabolism, participation in cutaneous homeostasis and the modulation of ultraviolet-induced damage (Furue et al., 2014). Some studies have linked the impact of *Malassezia* on skin diseases to its capacity to produce AhR ligands (Gaitanis et al., 2008; Magiatis et al., 2013). AhR ligands produced by *Malassezia* can possibly regulate the production of inflammatory mediators (Swanson, 2004) and/or change the function of keratinocytes (Vlachos et al., 2012). However, studies also highlighted the role of AhR ligands in host immunity (Lamas et al., 2018) on a more global view and on other types of cells; for example, AhR ligands are directly made by the gut microbiota from tryptophan transformation and have been connected recently to intestinal diseases such as IBD (Lamas et al., 2016; Agus et al., 2018). Consequently, the hypothesis of the involvement of *Malassezia* in gut pathologies via the AhR receptor should be investigated further.

### The Impact of Malassezia on Cancer

As mentioned above, there is an increasing number of clues suggesting that fungi can be implicated in the development of some cancers or at least that the mycobiota of CRC or CAC patients is modified. However, to date, very few specific fungi have been identified as central to these phenomena. Surprisingly, in the last 2 years, *Malassezia* strains have been independently identified as potentially key to cancer development in several publications.

Initial interest was obviously directed to gut-related cancer (CRC or CAC), and 2 recent works showed that the development of carcinoma was concurrent with the enrichment of *Malassezia* strains (Gao et al., 2017; Coker et al., 2019), suggesting a potential deleterious effect of this genus. In the first study, Gao and coworkers analyzed the mycobiota of colon polyps and CRC. Colorectal cancer is a malignant tumor in the colon and rectum, beginning with colon polyps that eventually evolve into carcinoma. There is strong evidence now that the gut bacteria composition plays a role in cancer development with a decrease in bacterial diversity and bacterial dysbiosis in favor of detrimental bacteria such as *Fusobacterium* spp. (Wong and Yu, 2019). Analyzing the mycobiota diversity of stool samples of patients with colon polyps, patients with colorectal cancer and healthy volunteers did not show any difference. However, the composition of the gut mycobiota revealed that the *Malassezia* genus was more abundant in patients with polyp and colorectal cancer. From these datasets, we can speculate that *Malassezia* strains can play a role in the genesis of colorectal cancer development, as they are present at the same level in precancerous lesions and in cancer.

In a recent work, Coker and collaborators performed a very interesting analysis on 3 large cohorts, 184 patients with CRC, 197 patients with adenoma and 204 control subjects, and showed that the mycobiota associated with CRC was specifically altered and might be responsible for triggering or amplifying colon adenoma (Coker et al., 2019). Using these data, they also showed that *Malassezia* was significantly increased in CRC, while *Saccharomycetes* was decreased, allowing the definition of potential efficient diagnostic markers for CRC prediction.

Finally, a study highlighted the role of mycobiota in the pathogenesis of pancreatic cancer (Aykut et al., 2019). The analysis of mycobiota in pancreatic patients showed an increased abundance of intrapancreatic fungi compared to healthy individuals, as well as in an associated mouse model. In pancreatic tumor tissue, the *Malassezia* genus was increased in both patients and mice. Furthermore, by administering GFP-labeled *S. cerevisiae* to mice, the authors demonstrated that large numbers of fungi migrate from the intestinal lumen to the pancreas. The authors highlighted the specific implication of *Malassezia* strains in the development of pancreatic lesions by ablating the mycobiota in mice using amphotericin B treatment and repopulating the gut with *M. globosa*; the genus accelerated the growth of pancreatic ductal adenocarcinoma tumors.

The gut-liver axis and the potential impact of microorganisms of the intestine on liver pathologies, such as non-alcoholic and alcoholic steatohepatitis, cirrhosis and hepatocellular carcinoma, could also be investigated, as the liver and intestine have a physical pathway via the portal vein and the bile duct (Alvarez-Silva et al., 2019).

## Conclusion

*Malassezia* can be associated with human gut-related disease. These fungi have been found in abundance in fecal samples, both in healthy and pathology contexts. Future studies designed to increase our understanding of *Malassezia* within intestinal dysbiosis as well as in other organs that can be connected to the gut may lead to novel therapeutic approaches that target this specific genus.

## Author Contributions

MS and MR constructed and wrote together the mini review.

## Conflict of Interest

The authors declare that the research was conducted in the absence of any commercial or financial relationships that could be construed as a potential conflict of interest.
